# RIG-I and MDA5 Protect Mice From Pichinde Virus Infection by Controlling Viral Replication and Regulating Immune Responses to the Infection

**DOI:** 10.3389/fimmu.2021.801811

**Published:** 2021-12-03

**Authors:** Morgan Brisse, Qinfeng Huang, Mizanur Rahman, Da Di, Yuying Liang, Hinh Ly

**Affiliations:** ^1^ Biochemistry, Molecular Biology and Biophysics Graduate Program, College of Veterinary Medicine, University of Minnesota, Twin Cities, MN, United States; ^2^ Department of Veterinary and Biomedical Sciences, College of Veterinary Medicine, University of Minnesota, Twin Cities, MN, United States

**Keywords:** Pichinde virus, mammarenavirus, arenavirus, innate immunity, RIG-I, MDA5, nucleoprotein

## Abstract

RIG-I and MDA5 are major cytoplasmic innate-immune sensor proteins that recognize aberrant double-stranded RNAs generated during virus infection to activate type 1 interferon (IFN-I) and IFN-stimulated gene (ISG) expressions to control virus infection. The roles of RIG-I and MDA5 in controlling replication of Pichinde virus (PICV), a mammarenavirus, in mice have not been examined. Here, we showed that MDA5 single knockout (SKO) and RIG-I/MDA5 double knockout (DKO) mice are highly susceptible to PICV infection as evidenced by their significant reduction in body weights during the course of the infection, validating the important roles of these innate-immune sensor proteins in controlling PICV infection. Compared to the wildtype mice, SKO and DKO mice infected with PICV had significantly higher virus titers and lower IFN-I expressions early in the infection but appeared to exhibit a late and heightened level of adaptive immune responses to clear the infection. When a recombinant rPICV mutant virus (rPICV-NPmut) that lacks the ability to suppress IFN-I was used to infect mice, as expected, there were heightened levels of IFN-I and ISG expressions in the wild-type mice, whereas infected SKO and DKO mice showed delayed mouse growth kinetics and relatively low, delayed, and transient levels of innate and adaptive immune responses to this viral infection. Taken together, our data suggest that PICV infection triggers activation of immune sensors that include but might not be necessarily limited to RIG-I and MDA5 to stimulate effective innate and adaptive immune responses to control virus infection in mice.

## Introduction

Mammarenaviruses are primarily rodent borne viruses, which are found in West Africa and South and Central America (1). Several members of this *Arenaviridae* family can cause hemorrhagic fever diseases in humans with limited options for treatment and vaccination (2–6). Lassa virus (LASV), which is an Old World mammarenavirus that causes endemic Lassa fever in several West African countries has been designated by the World Health Organization (WHO) among the blueprint priority pathogens that need the highest priority for research and development (7). Mammarenaviruses are transmitted *via* human contact with contaminated food and water or excreta from locally infected rodent populations [e.g., *Mastomys natalensis* (8, 9)] that do not appear to experience severe disease symptoms; and they therefore act as viral reservoirs (10–12).

There are currently no effective anti-viral treatments for Lassa fever other than the use of the non-specific anti-viral ribavirin, which must be given in the early stages of the disease to demonstrate efficacy (13). Except for the Candid #1 vaccine that has only been approved for use in Argentina to control human infections by the New World Junin virus (NW JUNV), there are currently no available vaccines for other pathogenic human mammarenaviruses. A notable factor in humans that has been shown to be associated with the development of severe disease due to LASV infection is the lack of the development of the proinflammatory cytokine responses in the early phase of the infection (14–17). Type 1 interferons (IFN-I), which consists of IFNα and IFNβ (18, 19), are critical cytokines needed to control the early phase of virus replication and to help initiate cellular inflammation as well as adaptive immune responses. Specifically, IFN-I expressions help drive the expression of hundreds of interferon-stimulated genes (ISGs) that play critical antiviral roles and aid the activation of T and B cells to produce cellular (helper CD4 and cytotoxic CD8 T cells) and humoral (antibody) responses against the virus infection (20). IFN-Is are expressed when the host RIG-I like Receptor (RLR) cellular proteins, which consist of RIG-I and MDA5, are activated upon recognizing pathogen-associated molecular pattern dsRNAs (PAMP dsRNAs) that are aberrantly generated during the process of viral RNA replication to activate the RLR signaling cascade to produce IFN-I (21, 22). While a significant amount of work has been done to elucidate the unique roles of RIG-I and MDA5 and their individual importance in antiviral defense (23), the overall and relative importance of each factor against mammarenaviral infection is not clear.

Pichinde virus (PICV), which is a NW BSL2-level mammarenavirus that normally does not cause disease in humans or mice but has been adapted for use in guinea pigs as a surrogate model of Lassa fever (24, 25). It adopts a life cycle like that of other mammarenaviruses. Virus particles enter cells by clathrin-mediated endocytosis (26), though the primary cellular receptor has not been identified for PICV (6). Replication occurs in the cytosol when the RNA-dependent RNA polymerase (L) in complex with the nucleoprotein (NP) synthesizes both anti-genomic and genomic strands of viral RNA, which is necessary for both viral replication and for viral gene expression (27). NP also has exoribonuclease (ExoN) functionality that is thought to degrade viral RNA to avoid detection by innate immune receptors, such as RIG-I and MDA5 (28). Formation and budding of progeny virus particles are aided by the viral Z matrix protein (29). While the host tropism of PICV has not been fully determined, the virus has been shown to infect both guinea pigs and mice as well as cells from several other species, including African green monkey (Vero) (30), hamster (BHK-21) (30) and human (PBMCs and THP1) (31). While the tissue tropism within mice and guinea pigs can be quite diverse, infectious virus and pathology mediated by virus infection can be found systemically in guinea pigs (25) and suckling mice (32, 33).

In the current study, we infected mice deficient for MDA5 alone (SKO) or both RIG-I and MDA5 (DKO) with PICV. We found that these KO mice experienced significant body weight loss and had significantly higher levels of infectious virus particles early in the infection that were cleared later during the course of the infection. These KO mice also had significantly depressed levels of IFN-I responses, but rather unexpectedly, appear to show similar levels of ISG expression as the WT mice and a heightened level of adaptive immune responses later in the course of the infection. Additionally, when a mutant PICV virus (rPICV-NPmut) that is known to be unable to suppress IFN-I expression was used to infect the WT as well as the SKO and DKO mice, all mice were able to effectively control virus infection, which further emphasizing the important roles of innate and adaptive immune responses that include but are not necessarily limited to RIG-I and MDA5, in controlling PICV infection in mice.

## Methods

### Plasmids and Cells

The pCAGGS mammalian expression vectors for generating PICV and PICV-NP-D380A (rPICV-NPmut) viruses have been described previously (34, 35). For expression and purification of the recombinant NP protein, the full-length NP gene was cloned into the pRSF-duet bacterial expression plasmid and contained a N-terminal histidine tag for expression in and purification from bacterial cells. Baby hamster kidney cells (BHK21) and African green-monkey kidney cells (Vero) were cultured in minimal essential media (MEM) supplemented with 10% heat-inactivated fetal bovine serum (FBS) and 50 μg/mL penicillin-streptomycin. BSRT7-5 cells, which were obtained from K.-K. Conzelmann (Ludwig-Maximilians-Universität, Germany), were cultured in minimal essential media (MEM) supplemented with 10% heat-inactivated fetal bovine serum (FBS) and 50 μg/mL penicillin-streptomycin.

### Virus Production and Sequencing

The methods for generating rPICV and rPICV-NPmut viruses have been described previously (35, 36). Supernatants containing these recombinant rPICVs were collected at 48 and 72 hours post PICV reverse genetics plasmids transfection. The rPICVs were amplified once in BHK21 cells, and the virus titers were determined by plaque assay. Viral RNA was extracted from viral stocks (or infectious viral plaques) using the QIAamp Viral RNA kit (Qiagen, USA), amplified by RT-PCR and sequenced to confirm the identity of the virus stocks. The lack of WT rPICV in the rPICV-NPmut virus stock was confirmed using a nested PCR reaction with internal primers specific to the WT and mutant sequences as described previously (35). Primer sequences used for viral sequencing reactions are provided in **Table 1**.

**Table 1:** Primer sequences used in PCR and RT-qPCR reactions.

Primer Name	Sequence
PICV NP PCR for mutation detection and sequencing primer- F	GGCATCAGCCAAGTCCTTTA
PICV NP PCR for mutation detection- R	TGTCTCAGCCTGGTGTTTATGG
Mouse ISG54 F	ATGAAGACGGTGCTGAATACTAGTGA
Mouse ISG54 R	TGGTGAGGGCTTTCTTTTTCC
Mouse ISG56 F	TGGCCGTTTCCTACAGIT
Mouse ISG56 R	TCCTCCAAGCAAAGGACTTC
Mouse ISG15 F	CTGAAGAAGCAGATTGCCCAGAAG
Mouse ISG15 R	CGCTGCAGTTCTGTACCACTAGC
Mouse IFNγ F	GAGGTCAACAACCCACAGGTC
Mouse IFNγ R	CGAATCAGCAGCGACTCCT
Mouse IFN ƛ F	AGCTGCAGGCCTTCAAAAAG
Mouse IFN ƛ R	TGGGAGTGAATGTGGCTCAG
Mouse ß-actin F	GGTCATCACTATTGGCAACG
Mouse ß-actin R	ACGGATGTCAACGTCACACT

### Mouse Lines and Handling

We used the approved protocol of the Institutional Animal Care and Use Committee (IACUC) at the University of Minnesota to conduct animal studies. MDA5 KO and MDA5/RIG-I DKO C57BL/6J mice were kind gifts of Dr. Michael Gale (University of Washington), and their generation has been described previously (37). WT C57BL/6J mice were obtained from the Jackson Laboratory (USA). Age-matched male and female mice between 6 and 20 weeks of age were infected with rPICVs *via* the intra-peritoneal (IP) route, and body weight was monitored daily following the infection. Blood was taken *via* the facial veins at various time points following the infection and used for analysis of IFNβ and ISG expressions, viremia, CD8+ T cells and antibody titers. At 3-, 6-, 9- and 15-days post infection, mice were euthanized, and organs and serum were collected for viral titer determination by plaque assay and for RNA extraction.

### Plaque Assay

Organs were prepared for plaque assay after tissue homogenization. Vero cells were seeded into 6-well plates at 60-70% confluence (4x10^5^ cells/well). In the following day, cells were infected with 500 µL of serially diluted rPICV stocks or of the organ homogenate in phosphate-buffered saline (PBS) for 1 hour at 37°C. After washing once with PBS, cells were incubated in MEM supplemented with 0.5% agar and cultured for an additional 4 days at 37°C and 5.0% CO_2_. Plaques were stained overnight with a diluted neutral red solution (0.01%) in 0.5% agar-MEM medium.

### Quantification of IFNβ Expression by ELISA

The IFNβ level in serum of the rPICV-infected mice was quantified using a mouse IFNβ enzyme-linked immunosorbent assay (ELISA) kit (LEGEND MAX™ Mouse IFNβ ELISA Kit with Pre-coated Plates) (Biolegend, USA) following the manufacturer’s instructions.

### RNA Extraction and RT-qPCR

Total RNA was extracted from spleens collected at 3- and 6-days post rPICV infection using TRIzol reagent (Life Technologies, USA). Single-stranded cDNAs were generated by the use of an oligo(dT) primer and MMLV reverse transcriptase (Promega, USA) following the manufacturer’s protocol. Quantitative RT-qPCR was conducted using specific primers designed to detect certain mouse genes of interest (**Table 1**) by using the IQ SYBR Green supermix (Bio-Rad, USA) in a Bio-Rad CFX quantitative PCR machine (Bio-Rad, USA) at 95°C for 3 min, followed by 40 cycles of DNA amplification at 95°C for 10 s and 60°C for 30 s. The data for each of the gene expressions were first normalized to the housekeeping gene β-actin and then compared to the level of the WT rPICV-infected mice using the 2^-ΔΔCT^ method.

### PICV NP Tetramer Analysis

Blood was collected from mice in lithium-heparin tubes for peripheral blood monocyte (PBMC) preparation. PBMCs were isolated from whole blood using lymphoprep separation technique (Fisher, USA) followed by lysis of any remaining red-blood cells using the RBC lysis buffer (Thermo-Fisher, USA). Cells were blocked with anti-mouse CD16/32 antibody (BD Biosciences, USA), and incubated with PE-labeled PICV NP(48-45)/H2K(b) MHC-I tetramer, provided by the NIH tetramer core facility at Emory University, together with CD8a-PerCP-Cy5.5 (eBioscience, USA), CD3-APC (Biolegend, USA), Zombie violet stain (Biolegend, USA), for 30 min on ice in the dark. After washing, cells were analyzed by flow cytometry on a BD FACSCelesta Cell Analyzer machine (BD Biosciences, USA). Data was analyzed with the FlowJo software (FlowJo LLC, USA).

### Anti-PICV NP IgG ELISA

pRSF-duet-P18NP was transformed into BL21 competent bacterial cells. After overnight IPTG induction (0.1 mM) at 16°C, the cells were lysed and the NP protein was purified on a HisTrap immobilized metal affinity chromatography (IMAC) column (Cytiva, USA). The Nunc MaxiSorp plates (Thermo-fisher, USA) were coated with 200 ng of recombinant NP proteins overnight at 4°C in coating buffer (50 mM Carbonate-Bicarbonate buffer, pH 9.5). Plates were washed once with wash buffer (PBS + 0.2% Tween20) and blocked for 2 h at ambient temperature with blocking buffer (PBS + 4.0% dry milk) followed by three more washes. Serum samples were serially diluted in diluting buffer (PBS + 0.05% Tween20), added to plates, and incubated for 1 h. Following three washes, samples were incubated with goat anti-guinea pig IgG HRP secondary antibody (Sigma, USA) at 1:10,000 dilution for 1 h. Following four washes, samples were incubated with the TMB substrate (Thermo-fisher, USA) for 15 min in the dark, then a stop solution (0.16M H_2_SO_4_) was added and optical density (OD) values were read at 450 nm using a Biotek Synergy 2 plate reader (Biotek, USA). The IgG antibody endpoint titer was defined as the highest dilution giving OD_450_ above the cutoff value, which was determined by average plus four times standard deviation of secondary-antibody-only controls.

### Statistical Analysis

All statistical analyses were performed using GraphPad Prism (GraphPad Software, USA). The statistical significance of the differences in the mean values was analyzed using an unpaired, two-tailed Student’s t test.

## Results

### MDA5 SKO and RIG-I/MDA5 DKO Mice Experience Significant Weight Loss During WT rPICV Infection

**Figure 1:**
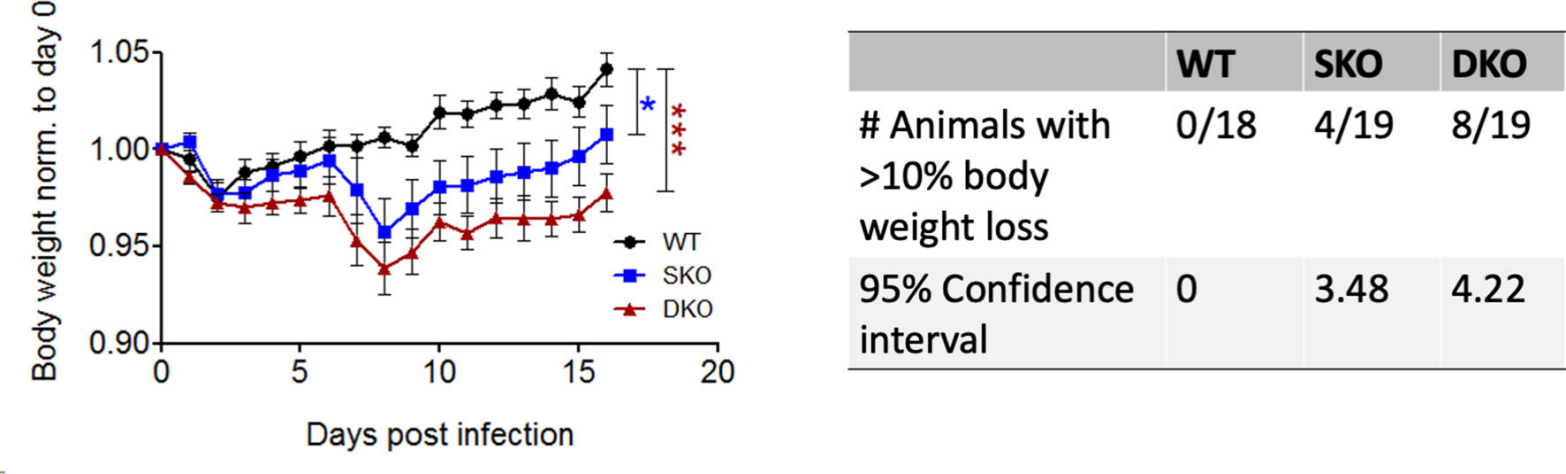
MDA5 KO and RIG-I/MDA5 DKO mice experience weight loss during rPICV infection. Body weight was measured daily for WT (n = 18), MDA5 SKO (n = 19) and DKO (n = 19) mice infected intraperitoneally (IP) with 1x10^5^ PFU of WT rPICV. The data are representative of 4 independent experiments. The significance values are reported as the level of statistical significance experienced each day between 2 groups after day 6 of infection when mice began to show weight loss. The number of animals in each group that experienced significant weight loss (defined as >10%) as well as the 95% confidence intervals for the proportion of animals that experienced significant body weight loss are reported in the table. *p < 0.05, **p < 0.01, ***p < 0.001.

To determine the importance of RIG-I and/or MDA5 against rPICV infection, body weight and morbidity data were collected daily for WT BL6, MDA5 KO and RIG-I/MDA5 DKO mice infected intra-peritoneally (IP) with 1.0x10^5^ PFU of the WT rPICV. While WT animals experienced no obvious signs of morbidity, 20% of the MDA5 SKO and almost 50% of the RIG-I/MDA5 DKO mice showed significant levels of body weight loss of at least 10% after day 6 post infection, but ultimately began to recover body weight after 9 days post infection (**Figure 1**). Notably, the overall changes in body weight were significantly different for the infected SKO and DKO mice as compared to the WT mice, but the differences were not significant between the infected SKO and DKO mice. These data suggest that the RIG-I and MDA5 innate immune pathway acts as a key barrier against rPICV infection in mice.

### RIG-I and MDA5 Are Essential for Controlling Early WT rPICV Replication in Mice

**Figure 2:**
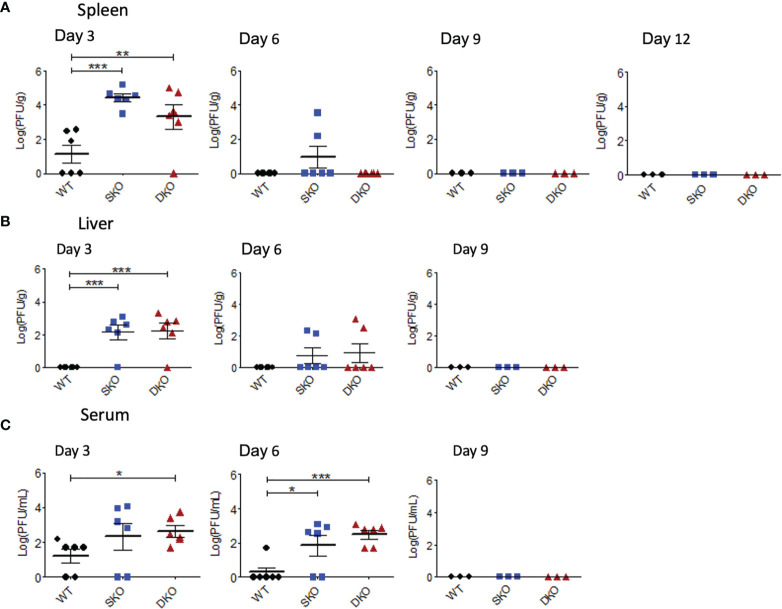
RIG-I and MDA5 are essential for controlling early WT rPICV replication in mice. rPICV infectious viral titers were determined by plaque assay and are reported for spleen **(A)**, liver **(B)**, and serum **(C)** from the infected mice at 3-day intervals following the infection. The data are representative of 2 independent experiments. *p < 0.05, **p < 0.01, ***p < 0.001.

RIG-I and MDA5 are most canonically known as early cellular protein sensors of viral infection and are critical for controlling viral replication (21). To establish a timeline of rPICV replication in mice and to examine the possible connectivity between morbidity and the levels of viral replication, mice infected with WT rPICV were sacrificed at various timepoints post infection to determine infectious viral titers in the spleen, serum, and liver by plaque assay. As previously observed in rPICV-infected guinea pigs (24, 25, 38), rPICV viral titer was highest in the spleen at day 3 post infection, which is thought to be due to the circulating monocytes trafficking into the draining lymph nodes being among the earliest targets of mammarenaviral infections (39, 40). Splenic virus titers were higher for the SKO and DKO mice as compared to the WT mice by several logs at day 3 post infection (**Figure 2A**) and were also significantly higher for both KO mouse lines in the liver (**Figure 2B**) and for the DKO mice in the serum (**Figure 2C**) at day 3 post infection. Notably, rPICV appeared to be cleared in the WT mice by day 6 and in the SKO and DKO mice by day 9 post infection, and while some virus titers were detected in a portion of the KO mouse lines in the spleen and liver at day 6, almost all KO mice had detectible viremia in the serum at day 6 (**Figure 2C**). Collectively, these data indicate that both RIG-I and MDA5 are essential for controlling early PICV replication in mice and that other cellular factors might be responsible for virus clearance later in the infection.

### WT rPICV-Infected MDA SKO and RIG-I/MDA5 DKO Mice Express Interferon-Stimulated Genes (ISGs) Despite a Seemingly Lack of Early IFN-I Signaling *via* the RIG-I and MDA5 Pathway

**Figure 3:**
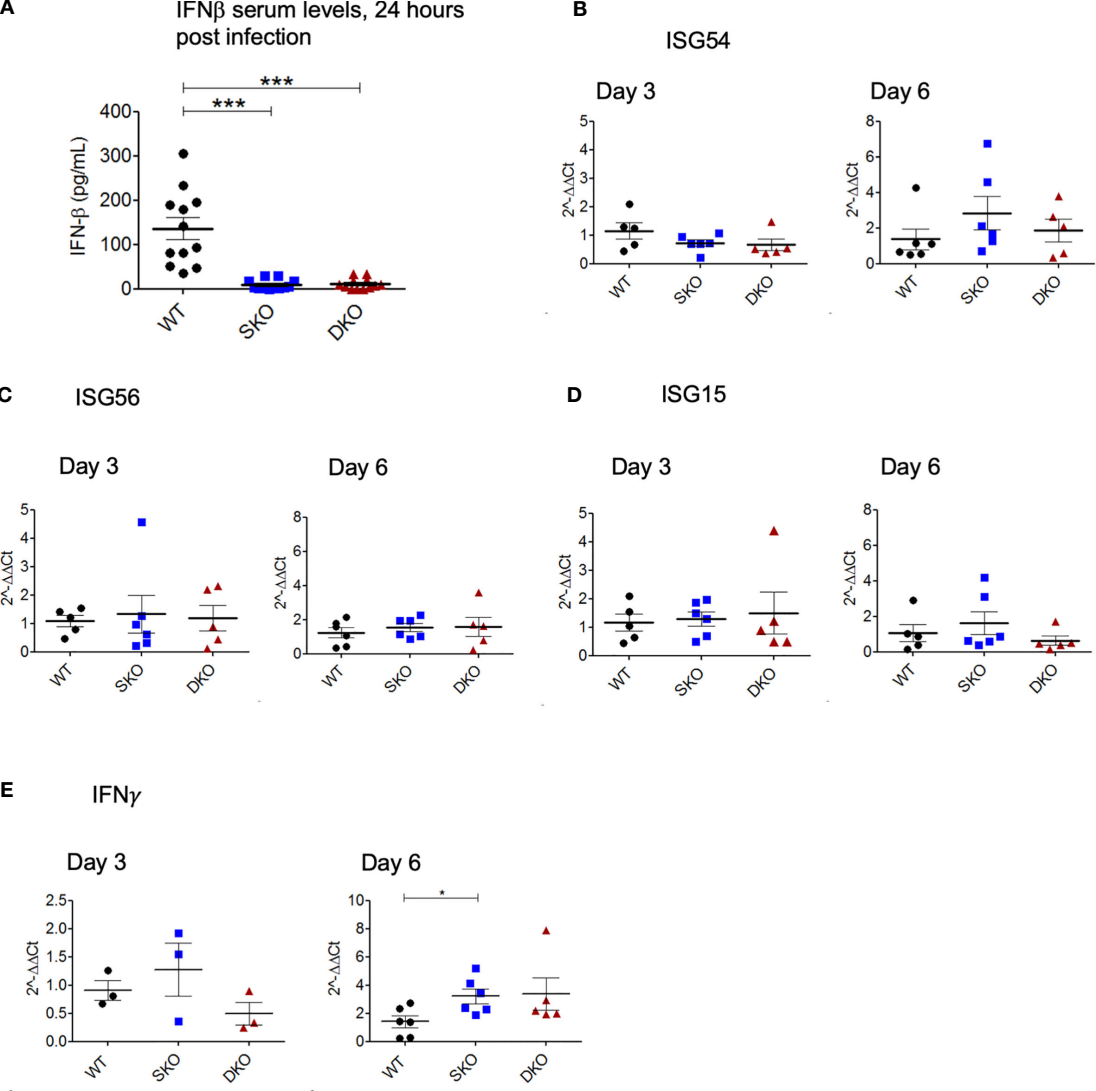
MDA5 SKO and RIG-I/MDA5 DKO mice have decreased early IFN-I signaling but express ISGs later in the course of the infection. Serum was isolated from whole blood taken 24 h after WT rPICV infection, and IFN-ß levels in the serum were determined by ELISA **(A)**. Total RNAs were extracted from spleens at 3 days and 6 days post WT rPICV infection and were analyzed by RT-qPCR to determine the expression levels of the ISG54 **(B)**, ISG56 **(C)**, ISG15 **(D)** and IFN-γ **(E)**. Data are representative of 2 independent experiments. *p < 0.05, **p < 0.01, ***p < 0.001.

We decided to first confirm whether RIG-I and MDA5 KO prevented the onset of an early IFN-I response to WT rPICV infection by performing ELISA on the serum taken 24 h post infection when IFNβ is known to reach peak systemic levels (41, 42). As expected, the rPICV-infected WT mice had significantly higher levels of serum IFNβ than the rPICV-infected SKO and DKO mice, indicating that these KO animals failed to launch a typical early and robust level of IFN-I expressions against rPICV infection (**Figure 3A**). Next, to test how the innate immune response progresses throughout the course of the WT rPICV infection, we quantified the gene expression levels of three well studied ISGs in splenic RNAs prepared at days 3 and 6 post infection by RT-qPCR. We measured the levels of gene expression of the ISG54, which is associated with cellular apoptosis (43), ISG56, which is known to be broadly inhibitive of cellular and viral replication (44), and ISG15, which induces proliferation and maturation of immune cells (45). RT-qPCR analysis showed no significant differences between rPICV-infected WT and SKO and DKO mice in ISG responses (**Figures 3B, D**), indicating that these two KO mouse lines were somehow able to express these particular ISGs by day 3 post viral infection despite the lack of early IFN-I signaling mediated by the RIG-I and MDA5 pathway. We also measured the levels of gene expression of the type II IFN (IFNγ) at days 3 and 6 post infection by RT-qPCR. There was also no significant difference in IFNγ expression at day 3, though there was a slightly significant increase in IFNγ expression in the SKO mice at day 6 (**Figure 3E**), the significance of which is not known. Taken together, these data indicate that while RIG-I and MDA5 are key regulators of IFN-I expression early during rPICV infection, mice lacking these cellular proteins may still be able to express a certain type of ISG genes during rPICV infection.

### WT rPICV-Infected MDA5 SKO and RIG-I/MDA5 DKO Mice Exhibited Heightened Levels of Adaptive Immune Responses to Virus Infection

**Figure 4:**
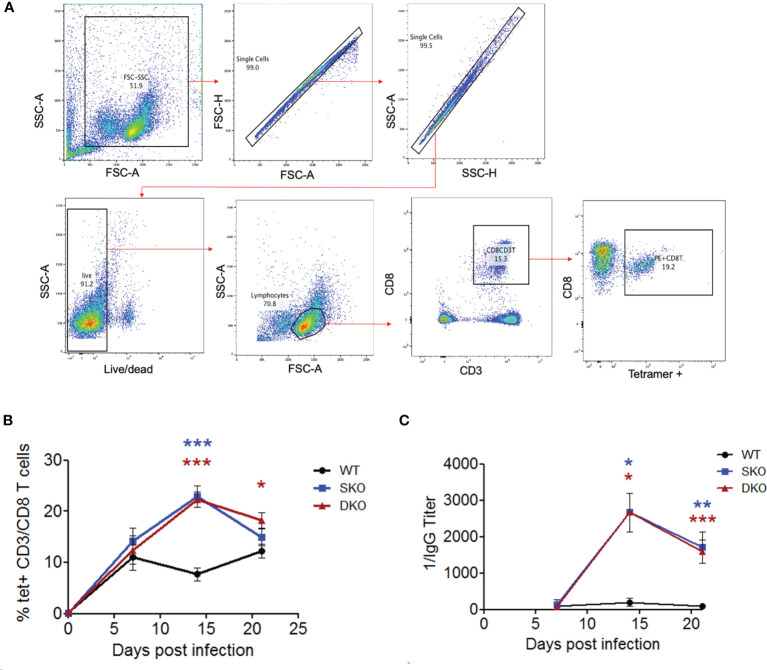
MDA5 SKO and RIG-I/MDA5 DKO increases adaptive immune responses to rPICV infection. **(A)** PBMCs were isolated from whole blood taken at 7 days, 14 days and 24 days post WT rPICV infection and were stained to analyze the levels of rPICV-specific CD8+ T cell responses. Cells were gated using the following gating strategy: whole cells (FSC-A/SSC-A), single cells (FSC-A/FSC-H, then SSC-H/SSC-A), live/dead (BV421/SSC-A), lymphocytes (FSC-A/SSC-A), CD8 T cells (APC-CD3/PerCP 5.5 CD8), P18 specific CD8 T cells (PE-tet/Percp 5.5 CD8). **(B)** The percentages (%) of CD8+ T cells that were also tetramer positive were reported at 7-day intervals during infection (n = 4). **(C)** Sera were isolated from whole blood taken at day 7, 14 and 21 post rPICV infection, and anti-PICV NP antibody titers were determined by ELISA. *p < 0.05, **p < 0.01, ***p < 0.001.

While not much is known about the specific roles of RIG-I and MDA5 in activating the adaptive immune responses to viral infection, IFN-I is well known to be critical for activating T and B cells to control virus infection (20, 46). Notably, effective adaptive immune responses [especially cellular immunity (15, 47)] have been shown to be critical for clearing LASV infection and for the Lassa fever recovery in humans (48, 49). We therefore decided to explore the potential impacts of RIG-I and MDA5 on the adaptive immune responses to WT rPICV infection of mice. First, we obtained PBMCs from the WT rPICV-infected mice at weekly intervals following virus infection and stained them with a known PICV-NP MHCI tetramer together with CD8+ T cell markers (**Figure 4A**). We found that the SKO and DKO mice developed stronger PICV-specific CD8+ T cells than WT mice, with the greatest levels of differences at day 14 post infection when tetramer+ CD8+ T cells peaked for both of the KO mouse lines (**Figure 4B**). We next quantified the levels of anti-PICV antibody response at days 7-21 post infection by ELISA and found that the anti-PICV antibody titers in both of the KO mouse lines were 1-2 log higher than those in the rPICV-infected WT mice at days 14 and 21 (**Figure 4C**). Taken altogether, these data indicate that despite the lack of early IFN-expressions mediated by RIG-I and MDA5, mice lacking these innate immune regulatory gene expressions are still able to mount effective adaptive immune responses against rPICV infection in mice.

### Absence of RIG-I and MDA5 Does Not Necessarily Abolish Anti-Viral Responses to Viral PAMP dsRNAs

One of the ways that mammaviruses evade the innate immune recognition upon virus infection is through the viral nucleoprotein (NP) exoribonuclease (ExoN) activity that can degrade PAMP dsRNAs (50–52). rPICV containing an ExoN-deficient NP has been shown to replicate at significantly higher levels in RIG-I KO mouse embryonic fibroblasts (MEFs) than in WT cells and can induce a strong level of IFN-I response in the WT cells (53).

**Figure 5:**
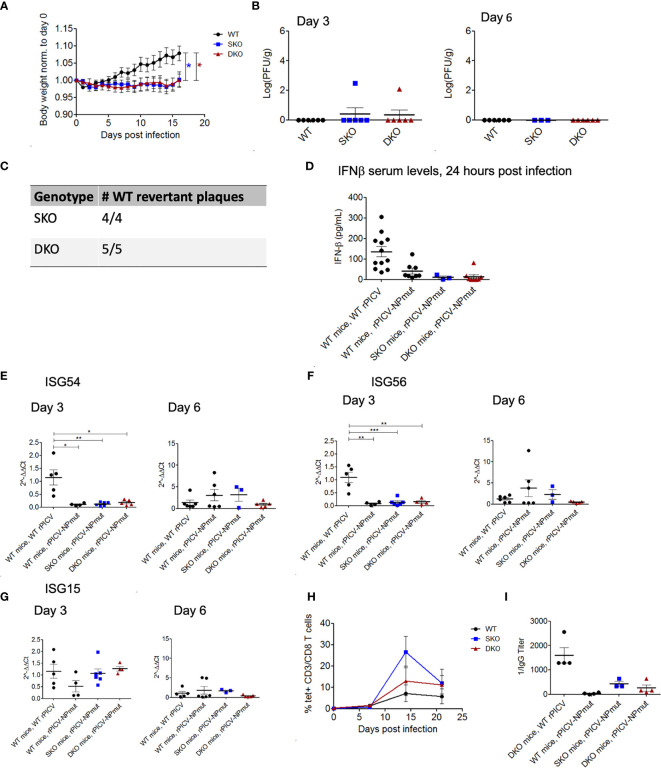
WT and KO mice robustly control rPICV-NPmut infection. **(A)** Body weight was measured daily for WT (n = 10), MDA5 SKO (n = 11) and RIG-I/MDA5 DKO (n = 11) mice infected intraperitoneally (IP) with 1x10^5^ PFU of rPICV-NPmut virus. Statistical significance was observed in comparing WT to SKO and DKO between days 12 and 16 post infection. **(B)** rPICV-NPmut infectious viral titers were determined by plaque assay and are reported for spleen at days 3 and 6 following rPICV-NPmut infection. **(C)** Total RNAs were extracted from individual viral plaque from some of the virus-infected spleens and were sequenced to confirm the identity of the intended Alanine substitution at residue 380 of the PICV NP gene of the rPICV-NPmut and of the NP WT-revertant with the Asparagine at this position. **(D)** Sera were isolated from whole blood taken 24 h after rPICV-NPmut infection, and the IFN-ß expression levels in the sera were determined by ELISA and compared to the IFN-ß expression levels seen with the WT mice infected with the WT rPICV. **(E–G)** Total RNAs were extracted from spleens taken 3- and 6-days post rPICV-NPmut infection and were analyzed by RT-qPCR to determine the expression levels of the ISG54, ISG56 and ISG15. **(H)** The percentages (%) of CD8+ T cells that were also tetramer positive were reported at 7-day intervals (n = 4). **(I)** Sera were isolated from whole blood taken at day 21 post rPICV-NPmut, and the levels of anti-PICV NP antibody were determined by ELISA. *p < 0.05, **p < 0.01, ***p < 0.001.

To determine the potential role of NP’s ExoN in rPICV-infected mice, we infected WT, MDA5 SKO, and RIG-I/MDA5 DKO mice with the rPICV-NPmut virus that contains a D380A mutation in NP gene. This D380A mutation has previously been shown to abolish the NP’s ExoN enzymatic activity and therefore can induce strong IFN-I responses in cell culture as well as in the rPICV-NPmut virus-infected guinea pigs (35). In the current study, none of the rPICV-NPmut-infected mice lose significant body weights during the course of the infection as compared to the rPICV-NPmut-infected WT mice (**Figure 5A**) with only two out of 21 SKO and DKO mice produced detectable levels of virus replication by plaque assay (**Figure 5B**). It is noteworthy that sequencing of the viruses recovered from those two rPICV-NPmut-infected SKO and DKO mice showed that all the viruses with the original NP D380A mutations have reverted to the WT sequence (**Figure 5C**), implicating the strong preference of the rPICV to maintain the NP’s ExoN function *in vivo*, even in the absence of a functional RIG-I and MDA5 immune surveillance system. Furthermore, whereas the WT mice infected with the WT rPICV produced high levels of early IFNβ and ISGs at day 3 post infection that were diminished by day 6 post infection (**Figures 5D–G**), none of the rPICV-NPmut-infected mice produced significant levels of these genes at both time points, except for a comparable level of ISG15 expression in the KO mouse lines at day 3 post infection as compared to that in the WT mice infected with the WT rPICV, the significance of which is not known.

Interestingly, rPICV-NPmut induced a delayed and transient T cell response in a subset of mice from all three genetic backgrounds. Specifically, the PICV-specific T-cells were only being detected starting at 14 days in the rPICV-NPmut-infected mice as compared to 7 days in the WT rPICV-infected mice. Additionally, the PICV-specific T-cells in the rPICV-NPmut-infected mice diminished by day 20 post infection (**Figure 5H**). Similarly, the anti-PICV antibody responses were significantly lower in the rPICV-NPmut-infected mice than in the WT rPICV-infected mice, with the strongest antibody responses observed in animals with the strongest T cell responses (**Figure 5I**). The differential levels of adaptive immune responses likely reflect the differences in the level of viral replication *in vivo*, as higher viral load generally induces stronger immune responses.

## Discussion

In this study, we have established the important roles of RIG-I and MDA5 as key innate-immune cellular factors for the natural protection of mice against PICV infection. This finding falls in line with previous studies of infection of RIG-I and MDA5 KO mice with other RNA viruses. For example, certain strains of the lymphocytic choriomeningitis virus (LCMV), an OW mammarenavirus, had been used in a variety of model organisms due to its ability to induce symptoms in immunocompetent animals that range from a lethal disease in rhesus monkeys (54) to a chronic infection in mice that is considered a good model to study T cell responses to infection and T cell exhaustion (55, 56). LCMV-infected MDA5 SKO mice demonstrated significantly lower IFN-I signaling than in the virus-infected WT mice (41, 42). They also had significantly higher splenic viral titers and delayed T cell responses up to 3 weeks as compared to the virus-infected WT animals (42). Similar findings were found using West Nile virus infection in MDA5 SKO mice (57) and in RIG-I SKO, MDA5 SKO and DKO mice (37) with notable differences in boosted IFN-I signaling in the MDA5 SKO mice (57). It was noted that RIG-I SKO mice infected with the influenza virus PR8 had significantly longer viral clearance times and lower numbers of activated T cells than the infected WT mice (58). Finally, it was found that mice treated with RIG-I-activating ligand prior to infection by SARS-CoV-2 had increased survivability, decreased viral titers and increased antibody responses as compared to the virus-infected WT mice, as described in a recent preprint (59). Taken together, these findings indicate that RIG-I and MDA5 consistently provide antiviral protection in mice by inducing early IFN-I expression to inhibit virus replication, as shown in our current study (**Figures 2**, **3**).

It is also notable that we could recover WT revertant PICVs from two of the mice infected with the rPICV-NPmut (**Figure 5C**). WT PICV revertants have also previously been recovered in the serum of guinea pigs infected with the same rPICV-NPmut (35), indicating that there is a strong selective pressure in both mice and guinea pigs to maintain a functional PICV NP ExoN for optimal virus replication in these animals. This is consistent with the fact that ExoN-deficient rPICV experiences significantly attenuated growth kinetics as compared to the WT rPICV in cell culture (35, 53). It is also important to note that the WT revertant PICVs were derived in the MDA5 SKO and RIG-I/MDA5 DKO mice (**Figure 5C**), which strongly suggests either an active immune pressure conferred by cellular factors besides RIG-I and MDA5 or the propensity of the virus to maintain an active NP ExoN function for optimal virus replication.

It is well documented that anti-viral activity must also rely on other innate immune sensors, such as the Toll-like receptors (TLRs) (60), protein kinase R (PKR) (61) and Laboratory of Genetics and Physiology protein 2 (LGP2) (62), all of which can also recognize either PAMP RNAs or various other aspects of the pathogens upon virus infection. In the context of mammarenaviral infection, activation of these innate immune receptors has been documented, in particular, with the NW Junin virus (JUNV) infection, in which a strong and sustained inflammatory response is likely associated with the development of severe disease (6, 63, 64). The TLR2/6 complex has been shown to be critical for antiviral activity against JUNV infection (65, 66). Likewise, PKR has been found to be activated upon JUNV infection in cell culture, but it did not have a significant impact on virus replication (67). While it is possible that the innate immune responses initiated by PICV infection may be similar to JUNV and other NW mammarenavirus infections, further studies are needed to fully understand the immune responses to PICV infection in comparison to other pathogenic vs. non-pathogenic mammarenaviruses of both NW and OW groups, and in different experimental systems.

It is also possible that expression of other members of the interferon family contributed to the expression of ISGs in the KO mice. The IFN-I family consists of several members, the most predominant being IFNα and IFNβ but also including others, such as IFNκ, IFNω and IFNτ (68). It is noteworthy that ISG expressions can also be stimulated by the type II (IFNγ) and type III (IFNΛ) interferons (69–71). Some studies have characterized the expression of type II interferons during mammarenaviral infection (72–78). In the current study, we found no significant difference in IFNγ expression in day 3 and a slightly significant increase of IFNγ expression in the SKO mice at day 6. This may be correlated with the strengthened CD8 T cell responses in these mice given the well characterized role of IFNγ in initiating T cell growth (79) and its subsequent expression by activated T cells (70). However, we did not find any quantifiable expression of IFNΛ in the spleen of infected mice (data not shown). This is consistent with previous finding of IFNΛ being most highly expressed in mucosal organs, such as the lungs, but at very low levels in the spleen (80). Unfortunately, no lung samples were preserved for the IFNΛ analysis in our study. Regardless, we believe that type II (but not likely type III) interferons may play an important role in mediating splenic ISG expressions in the KO mice.

We also noted a higher level of adaptive immune responses to rPICV infection in the SKO and DKO mice than in the WT mice (**Figures 4B, C**). It is possible that a relatively small level of IFN-I expressions (**Figure 3A**) in these genetically altered mice in response to an active virus replication (**Figure 2**) can still trigger robust levels of ISG expressions (**Figures 3B–D**) and cellular immunity (**Figure 4**). A stronger level of adaptive immune responses in the SKO and DKO mice at the later timepoints during the infection strongly indicates that both humoral and adaptive immune responses might be necessary to fully control the infection in these mice. This data falls in line with early studies showing potent T and NK responses in PICV infected mice (81, 82) that were critical for clearing viral infection (83). Adaptive immunity to PICV infection is also relevant considering that cellular immunity have been shown to be essential for clearing LASV infection (15, 47) and in preventing the development of serious disease (48, 49).

Apart from the data showing that more DKO mice experiencing significant weight loss than the SKO mice during WT rPICV infection, we did not observe any other significant differences between these genetically altered mouse lines. However, previous studies have suggested that RIG-I is more important in controlling replication of many known negative-sense RNA viruses, including mammarenaviruses (53, 84, 85), whereas MDA5 is important in controlling many positive-sense RNA virus infections (21, 23). This is likely due to the differences in the RNA ligands that these viruses produce during the course of the infection, with RIG-I mostly responding to short, double-stranded phosphorylated RNAs, and MDA5 responding to long double-stranded RNAs (21). However, in West Nile virus infection (86), RIG-I and MDA5 appear to have an additive anti-viral effect, whereas either of these two cellular proteins can provide protection against Dengue virus infection (87). Such complexities are likely driven by either the differing species of the PAMP RNAs generated during virus infections or other aspects of the viruses (e.g., viral lipids or proteins) that can also trigger IFN expressions *via* TLR activations, for instance.

In summary, we have demonstrated in the current study that RIG-I and MDA5 are important in providing protection against rPICV infection in mice by controlling the early stages of viral replication through IFN-I activation. Our data also suggest that RIG-I and MDA5 might not be the only cellular factors responding to rPICV infection but that activation of adaptive immunity may also be necessary to effectively clear the infection. Further studies are needed to understand the full spectrum of immune sensors and responses to PICV and other mammarenavirus infections.

## Data Availability Statement

The original contributions presented in the study are included in the article/supplementary material. Further inquiries can be directed to the corresponding author.

## Ethics Statement

The animal study was reviewed and approved by University of Minnesota Institutional Animal Care and Use Committee.

## Author Contributions

The authors confirm contribution to the paper as follows. Study conception and design: MB, HL, and YL. Data collection: MB, QH, MR, and DD. Analysis and interpretation of results: MB, HL, and YL. Draft manuscript preparation: MB, HL, and YL. All authors reviewed the results and approved the final version of the manuscript.

## Funding

This work was supported in part by the NIH NIAID grant R01 AI131586 to HL and YL and by a predoctoral NIH fellowship T32 DA007097 and the University of Minnesota Doctoral Dissertation Fellowship (DDF) to MB.

## Conflict of Interest

The authors declare that the research was conducted in the absence of any commercial or financial relationships that could be construed as a potential conflict of interest.

## Publisher’s Note

All claims expressed in this article are solely those of the authors and do not necessarily represent those of their affiliated organizations, or those of the publisher, the editors and the reviewers. Any product that may be evaluated in this article, or claim that may be made by its manufacturer, is not guaranteed or endorsed by the publisher.
